# Moyamoya disease presenting with tubular dysfunction in a child: pitfalls in diagnosing an atypical hyponatremic-hypertensive syndrome

**DOI:** 10.1186/s12887-023-03926-1

**Published:** 2023-05-08

**Authors:** Maria Luisa Conte, Claudio La Scola, Francesca Mencarelli, Beatrice Filippini, Elena Fabbri, Valentina Ragnoni, Elisa Ravaioli, Andrea Pasini, Gianluca Vergine

**Affiliations:** 1 Department of Pediatrics, Infermi Hospital, Viale Settembrini, 2, 47900 Rimini, Italy; 2 Pediatric Nephrology and Dialysis, Pediatric Unit, IRCCS AziendaOspedaliero-Universitaria Di Bologna, Bologna, Italy

**Keywords:** Hyponatremic-hypertensive syndrome, Kidney tubular dysfunction, Unilateral renal artery stenosis, Moyamoya disease, Children

## Abstract

**Background:**

Moyamoya disease, a cause of pediatric stroke, has been shown to affect furthermore extra-cranial districts, mostly the kidney arterial site, resulting in steno-occlusive changes. Unilateral renal artery stenosis accounts for 8%-10% out of cases of renovascular hypertension in childhood, however it rarely underlies a hyponatremic-hypertensive syndrome (HHS).

**Case presentation:**

We describe an 18-month-old boy with a recent history of polyuria and polydipsia, who presented an acute febrile gastroenteritis with neurological impairment, severe dehydration, hyponatremia, hypokalemia, kidney tubular dysfunction, and elevated aldosterone and renin even with a normal blood pressure. Fluid and electrolytes correction was performed, with complete recovery. An abdominal ultrasound displayed a smaller right kidney. A brain magnetic resonance and an electroencephalogram did not show any relevant abnormalities. Five months later, the child experienced a left-side hemiparesis after a traumatic concussion, and a severe hypertension. A brain tomography documented a cerebral ischemia. Brain and kidney angiographic studies displayed *puff of smoke* findings of internal right carotid artery branches and a steno-occlusive pattern of right renal artery, respectively. Hence, moyamoya disease with HHS secondary to unilateral renal artery stenosis was diagnosed. After an unsuccessful antiplatelet and antihypertensive pharmacological treatment, the boy underwent a renal angioplasty and a cerebral STA-MCA bypass (direct superficial temporal artery-to-middle cerebral artery bypass), resulting in a significant improvement of both neurological and kidney disease.

**Conclusions:**

Although the association between unilateral renal artery stenosis and HHS has been previously shown, this is the first report of atypical HHS, with hypertension preceded by tubular dysfunction, recognized in the framework of moyamoya disease.

## Background

Moyamoya disease is a progressive steno-occlusive cerebrovascular arteriopathy of unknown origin involving the distal intracranial internal carotid artery and its main branches, and is a rare cause of pediatric stroke. Hallmark of moyamoya disease is the presence of a collateral network of overgrown and dilated small arteries, originating from the circle of Willis, which appear as a *puff of smoke*, thus the Japanese term moyamoya [1]. In about one-third of moyamoya pediatric cases, the distinctive angiographic images are found in certain conditions (neurofibromatosis type 1, sickle cell disease, Down syndrome, autoimmune diseases) and such patients are preferably diagnosed as moyamoya syndrome instead of moymoya disease [2].

Clinical features of moyamoya disease differ between children and adults. According to the International Pediatric Stroke Study, initial presentation of moyamoya in children is ischemic stroke in 90% of cases, transient ischemia in 7.5% and hemorrhagic stroke in 2.5%, with hemiparesis as the most common presenting symptom [3]. In contrast, about half of adult patients develop intracranial bleeding [4]. Rate of epilepsy is also higher in children than in adults [5].

In patients with moyamoya disease, steno-occlusive arterial changes may likewise encompass extra-cranial arteries, as well as carotid, pulmonary, coronary, and renal arteries [6].

Herewith, we describe an unusual and insidious case of moyamoya disease in an 18-month-old child presenting with HHS, related to unilateral renal arterial stenosis, with no hypertension at onset. To our knowledge, no association between moyamoya disease and HHS has been previously described. Moreover, HHS has never been reported as onset of a moyamoya disease.

## Case presentation

**Fig. 1:**
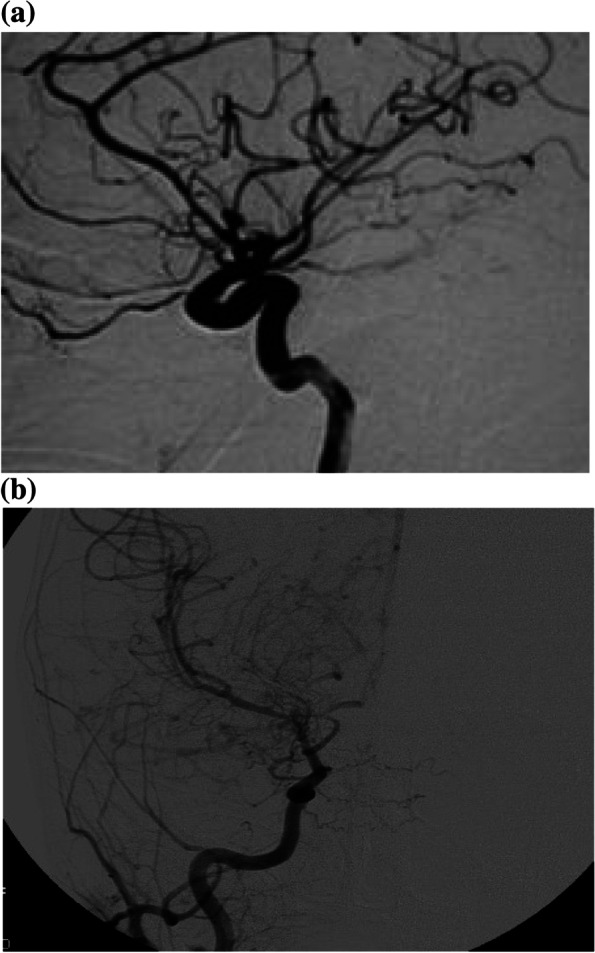
Cerebral angiography, showing a right internal carotid artery stenosis with *puff of smoke* pattern of collateral vessels in lateral (**a**) and anterio-posterior (**b**) view (Moyamoya disease)

The boy was born at 34 weeks of gestational age, after a pregnancy complicated by impending abortion, with a birth weight of 3075 g, not experiencing asphyxia, hypoglycemia, or other perinatal complications. Family history disclosed hypertension, kidney asymmetry and myocardial infarction at the age of 50 years in paternal grandfather, and hypertension in a paternal aunt. A febrile urinary tract infection occurred at age of 6 months. At that time, a kidney ultrasound revealed a slight renal asymmetry (right kidney 47 mm, left kidney 56 mm) and follow-up urine tests showed intermittent borderline proteinuria (maximum Upr/Ucr 0.5 mg/mg); instead, plasma creatinine and blood pressure were normal. At the age of 18 months, he was admitted to the hospital because of dehydration during an acute febrile viral enteritis. A history of polyuria and polydipsia, noticed during the first year of life and afterwards worsening, was disclosed by parents. Physical examination on admission showed severe dehydration, irritability, hypotonia and drowsiness. Body weight was 13 kg (81^th^ percentile), height was 96 cm (> 95^th^ percentile) and head circumference 51 cm (> 95^th^ percentile) according to CDC charts. Blood pressure was 90/60 mm Hg (< 90^th^ percentile according to AAP Guidelines 2017) [7]. Laboratory blood examination showed metabolic alkalosis (pH 7.56, HCO3^−^ 30.3, BE -8.1), hemoglobin 17.5 g/dL, Hct 46.5%, white blood cells 5290/μL (neutrophils 1390/μL), platelets 88 × 10^3^/μL, creatinine 0.37 mg/dL, eGFR 142 mL/min/1.73 m^2^ (Schwartz equation, k = 0.55), plasma osmolality 292 mOsm/Kg (normal values (n.v.) 275–300), Na^+^ 123 mMol/L (n.v. 136–145), K^+^ 2.9 mMol/L (n.v. 3.5–5.1), phosphates 3.6 mg/dL (n.v.2.5–6.4), uric acid 1.9 mg/dL (n.v. 3.4–7), renin 210 mUI/L (n.v. 4.4–46 mUI/L) and aldosterone > 1000 ng/L (n.v. 35–300 ng/L). The tubular function test showed an increased fractional excretion of sodium (2.6%, n.v. 0.5%-1.5%) and uric acid 26%, (n.v. < 20%), a reduced tubular reabsorption of phosphate (TRP 77%, n.v. > 85%), hypercalciuria (Uca/Ucr 1, n.v. for age < 0.5 mg/mg) and proteinuria (Upr/Ucr 30, n.v. for age < 0.5 mg/mg). The tubular potassium gradient was mildly augmented (TTKG 11, n.v. 5–10), urine osmolality was 237 mOsm/Kg H_2_O (n.v. 300–800), urine pH and specific gravity were 8 and 1006, respectively, and α-1-microglobulin 0.008 g/L (n.v. < 0.010). Increased plasmatic level of aldosterone, metabolic alkalosis and increased TTKG suggested a secondary hyperaldosteronism because of volume depletion. During hospitalization polyuria was present despite dehydration (about three liters/day), and an abdominal ultrasound confirmed the kidney asymmetry (right kidney 65 mm, left kidney 83 mm). A neurological consult showed a mild neurodevelopment delay. To investigate the persistence of irritability with onset of ataxic gait, the child underwent an electroencephalogram, which was normal, and a brain magnetic resonance that excluded either parenchymal abnormalities or evidence of ischemic damage. After the correction of the hydro-electrolyte disorders, the neurological symptoms improved, and the tubular function normalized in a few days (Table 1). Hence, the child wasdischarged, and in the following few weeksshowed again the borderline intermittent proteinuria (maximum Upr/Ucr 0.5 mg/mg). Five months later, at the age of 23 months, the boy was admitted to a foreign hospital for vomiting, left-sided hemiparesis, and obtunded sensorium after a head occipital trauma. A brain tomography displayed a right cerebral ischemia. At same time, a severe hypertension was detected (160/100 mmHg), poorly controlled by a combined treatment with ACE-inhibitor (enalapril), beta-blocking (carvedilol) and Ca^++^-antagonist (nifedipine, then amlodipine). A cardiac ultrasound showed concentric hypertrophy of the left ventricle, with normal electric conduction. A *fundus oculi* excluded hypertensive retinopathy. Laboratory testing showed normal creatinine and electrolytes, and an over-activation of renin–angiotensin–aldosterone axis (Table 1). A thrombophilia and an autoimmune underlying disease were excluded (data not shown). After the referral of the boy to our hospital, a cerebral and renal angiographic study was performed, revealing both aright internal carotid artery stenosis (70–80%) with compensatory vascular pathways displaying a *puff of smoke* pattern, and a severe right renal artery stenosis (95% at the distal tract of vessel). According to the representative angiographic brain findings (Fig. 1), moyamoya disease was diagnosed, together with a renovascular hypertension secondary to renal artery occlusion.

**Table 1:** Blood pressure and biochemical parameters in our patient

**TIME**	**Day 1**	**Days7-9**	**5 Months Later**
Blood pressure			
systolic (mm Hg)	90	95	160
diastolic (mm Hg)	60	60	100
**BLOOD**			
osmolality (mOsm/L)	292	280	283
sodium (mMol/L)	123	140	139
potassium (mMol/L)	2.9	3.9	4.7
chloride	95	106	103
phosphate (mg/dL)	2.5	4.2	4.4
uric acid (mg/dL)	1.9	3.9	2.2
creatinine (mg/dL)	0.3	0.34	0.5
renin (mUI/L)	210	286	1622 (n.v. 2.8–39.9 mUI/l)
aldosterone (ng/L)	> 1000	> 1000	379 (n.v. 30–300 pg/ml)
pH	7.56	7.4	7.43
bicarbonate (HCO3^−^)	30.3	26	26
base excess (BE)	8.1	2.5	2.2
**URINE**			
osmolality (mOsm/L)	237	350	296
Fe Na% (n.v. 0.5–1.5%)	2.6	0.8	0.9
Fe uric acid % (n.v. < 20%)	26	9.5	8
TRP% (n.v. > 85%)	77	95	90
calcium/creatinine	1.9	0.29	0.02
protein/creatinine	20	0.35	0.3
TTKG (n.v. 5–10)	11	8	2.2

*FeNa%* fractional excretion of sodium, *Fe uric acid %* fractional excretion of uric acid, *TRP%* tubular phosphate reabsorption, *TTKG%* transtubular K gradient, *n.v.* normal values

**Fig. 2:**
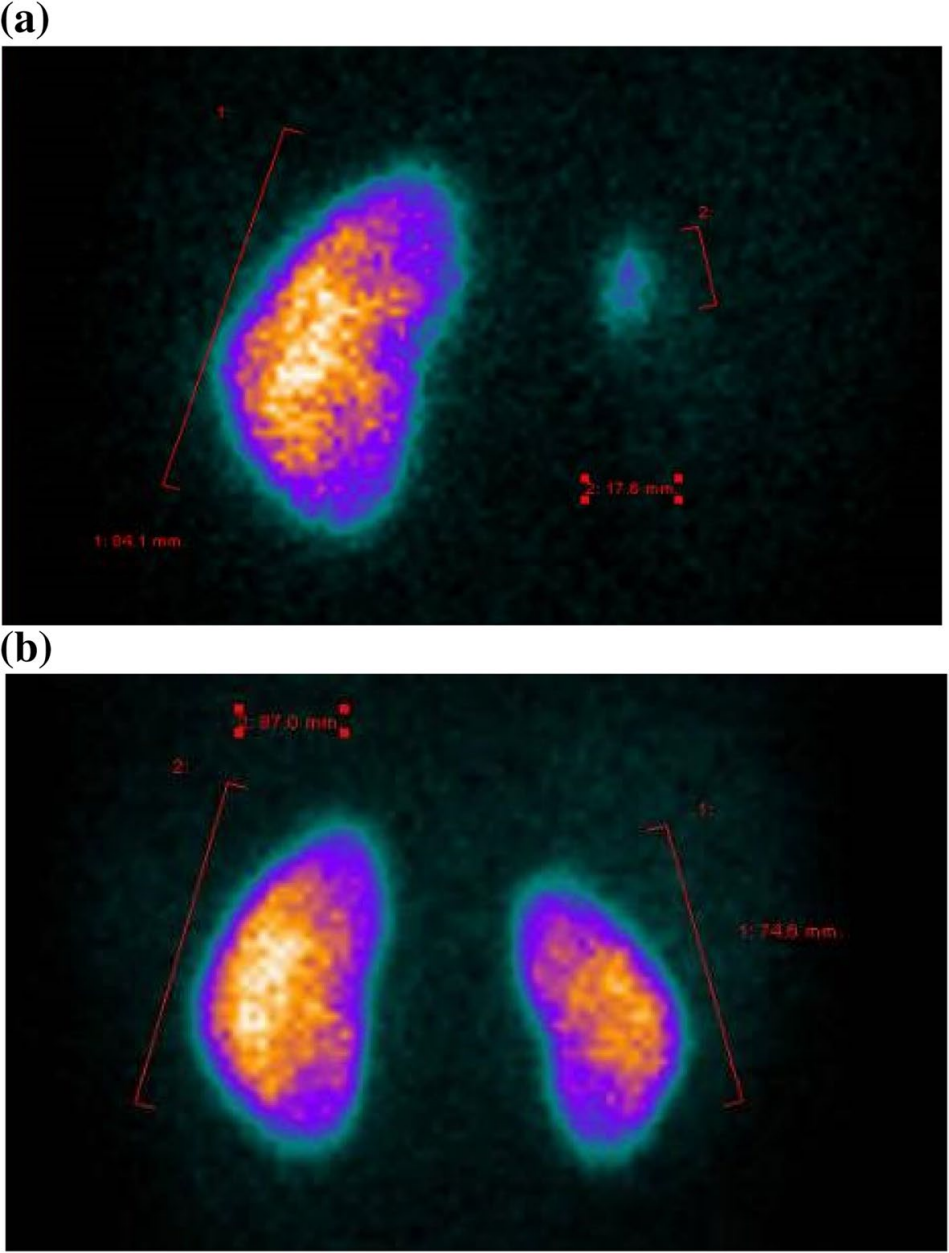
Tc-99 m DMSA scan (dimercaptosuccinic acid labeled with Technetium-99 m) before (**a**) and after (**b**) right renal artery angioplasty

The child was given acetylsalicylic acid and anti-hypertensives (atenolol, clonidine and nifedipine) with improvement but not normalization of blood pressure (120/70 mmHg, > 95^th^ percentile according to AAP Guidelines 2017). A second transient cerebral ischemic event occurred, worsening the left-sided neurological impairment. Consequently, in a foreign high-specialized institution, renal angioplasty and a cerebral STA-MCA bypass (direct superficial temporal artery-to-middle cerebral artery bypass) were conducted successfully, followed by a proper rehabilitative plan. The hemiparesis improved until complete resolution, and a excellent control of hypertension with amlodipine and atenolol was achieved. A kidney scintigraphy (Tc-99 m DMSA scan), performed before and after the surgical revascularization, displayed a quite impressive improvement of right kidney function (Fig. 2).

In order to define the etiology of moyamoya disease, we underline that clinical signs of syndromes associated with moyamoya angiopathy like neurofibromatosis and down syndrome were not found. Furthermore, the clinical history and laboratory investigations allowed us to exclude a sickle cell disease, other possible associated disease. Genetic test for moyamoya associated gene mutations has not yet been performed.

## Discussion and conclusion

Here we describe a child with moyamoya disease diagnosed several months after a HHS, due to unilateral renal artery stenosis and unusually without hypertension at onset.

HHS, a clinical entity wherein unilateral renal artery steno-occlusion drives hyponatremia and hypertension, acknowledges a pathogenetic activation of renin–angiotensin–aldosterone system and it has been found in about 16% of adults with unilateral renal artery stenosis [8], but rarely in pediatric patients [9, 10]. In detail, a renin over-secretion enhanced by renal ischemia, accounts for high circulating angiotensin II levels and aldosterone release. Hyperaldosteronism leads to increased blood volume expansion, hypertension, and potassium depletion. Angiotensin II induces a glomerular hyperfiltration on the non-stenotic kidney, responsible for wide loss of solutes and water, thus resulting in proteinuria, hypokalemia, hyponatremia, hypercalciuria, glycosuria and polyuria. Wasting of sodium and hypovolemia lead to a further stimulation for the renin release, together with the hypokalemia due to urine loss of potassium caused by secondary hyperaldosteronism. Reduction of circulating blood volume, on its behalf, worsens hyponatremia, by increasing the anti-diuretic hormone secretion [11]. Moyamoya arteriopathy, an intracranial vascular steno-occlusive illness causing neurological impairment, presents as ischemic stroke in 90% of pediatric cases [3]. Besides, several case-series reported extra-cranial vascular steno-occlusive changes [4, 6, 12, 13]. Among these, the renal artery is the most common site of vascular involvement, recently reported in eight out of 101 (7,9%) pediatric patients with moyamoya disease [13]. Interestingly, it should be noted that, in moyamoya disease, arterial changes are progressive and, moreover, a de novo renal artery stenosis might develop in originally *normal* arteries during a long-term follow-up [14].

The most remarkable finding of our patient is that tubulo-interstitial abnormalities preceded the development of hypertension, similarly to only three pediatric cases recently reported [11, 15, 16].

Instead, many other authors described adult and pediatric cases of HHS with typically severe hypertension at onset, associated with hyponatremia and tubular dysfunction [9, 11]. It is not completely elucidated why only in a few pediatric cases of HHS, included this one, hypertension is not noticed at onset of illness. Since arterial occlusive changes in moyamoya disease progress and worse with time, we speculate that acute phase of HHS might firstly display tubular dysfunction because, in initial stages of renal artery stenosis, hyperfiltration may be predominant over the hypertensive response to angiotensin, which develops later, only when stenosis becomes *critical*. Accordingly, in our patient, a severe dehydration enhanced by a viral infection might have triggered an HHS by acting on a kidney with *initial* arterial changes. The concurrent remark of renal asymmetry and intermittent mild proteinuria, expected consequence of renal arterial stenosis, supports our hypothesis. We highlight that any natriuretic-hyponatremic condition along with tubular impairment and kidney size asymmetry, even without early hypertension, may underlie a progressive renovascular disease and thus deserves a close blood pressure monitoring.

In conclusion, we report the first case of atypical hyponatremic hypertensive syndrome as onset of moyamoya arteriopathy in a child, with hypertension following tubular dysfunction and driven by unilateral renal artery stenosis.

## Data Availability

The data used to support the findings of this study are available from the corresponding author upon request.

## Abbreviations

HHS: Hyponatremic-hypertensive syndrome
STA-MCA bypass: Superficial temporal artery-to-middle cerebral artery bypass
DMSA: Dimercaptosuccinic acid
AAP: American Academy Pediatrics
CDC: Centers for Disease Control
TRP: Tubular reabsorption of phosphate
TTKG: Transtubular potassium gradient
